# MKK4 Knockdown Plays a Protective Role in Hemorrhagic Shock-Induced Liver Injury through the JNK Pathway

**DOI:** 10.1155/2022/5074153

**Published:** 2022-09-17

**Authors:** Hao Yao, Yu Gao, Jiahui Han, Yan Wang, Jimin Cai, Yongjun Rui, Xin Ge

**Affiliations:** ^1^Department of ICU, Wuxi 9th People's Hospital Affiliated to Soochow University, Wuxi, Jiangsu 214000, China; ^2^Department of Traumatic Orthopedics, Wuxi 9th People's Hospital Affiliated to Soochow University, Wuxi, Jiangsu 214000, China

## Abstract

Hemorrhagic shock (HS) triggers tissue hypoxia and organ failure during severe blood loss, and the liver is sensitive to HS. Mitogen-activated protein kinase kinase 4 (MKK4) activates the c-Jun NH2-terminal kinase (JNK) pathway, and its expression is upregulated in the serum of HS patients and mouse livers at 1 h post-HS. However, the function of MKK4 in HS-induced liver injury is unclear. The role of MKK4 was investigated *in vivo* using rat models of HS. Before HS, lentivirus harboring shRNA against MKK4 was injected into rats via the tail vein to knock down MKK4 expression. HS was induced by bloodletting via intubation of the femoral artery followed by resuscitation. The results showed that MKK4 knockdown reduced HS-induced apoptosis in the liver by decreasing Bax expression and the cleavage of caspase 3 and promoting Bcl-2 expression. Moreover, the generation of intracellular reactive oxygen species (ROS) and malondialdehyde (MDA) in the liver was promoted, while superoxide dismutase (SOD) activity was inhibited by HS. However, the effect of HS on oxidative stress was abrogated by MKK4 knockdown. Furthermore, MKK4 knockdown restored MMP and complex I and complex III activities and promoted ATP production, suggesting that HS-induced mitochondrial dysfunction in the liver was ameliorated by MKK4 knockdown. The inhibitory effect of MKK4 knockdown on the phosphorylation and activation of the JNK/c-Jun pathway was confirmed. Overall, MKK4 knockdown may suppress oxidative stress and subsequent apoptosis and improve mitochondrial function in the liver upon HS by inhibiting the JNK pathway. The MKK4/JNK axis was shown to be a therapeutic target for HS-induced liver injury in this study.

## 1. Introduction

Hemorrhage is the leading cause of death after trauma [1, 2]. Hemorrhagic shock (HS) is a life-threatening condition related to decreased blood flow and oxygen delivery and is characterized by tissue hypoxia and inflammation. HS and subsequent recovery can induce organ ischemia/reperfusion injury (IRI) [3]. Patients who survive this initial phase of HS remain likely to suffer from systemic inflammation and multiorgan failure even if they are adequately supplemented with fluids [4, 5]. The liver, which is sensitive to HS-induced injury, is considered one of the first organs attacked by hypoxia upon HS. Up to 5% of patients who survive 24 h after HS may ultimately be subjected to hepatic failure [6–8].

Mitogen-activated protein kinase kinase 4 (MKK4), belonging to the MAP kinase kinase family, is a kinase that directly phosphorylates c-Jun NH2-terminal kinase (JNK) [9]. MKK4, which was the first kinase identified that specifically and directly phosphorylates JNK at tyrosine 185, is essential for optimal JNK activation [10, 11]. JNKs are stress-activated protein kinases. The JNK signaling pathway, which is one of four MAPK cascades, can be activated by MKK4 in response to diverse oxidative stress-induced stimuli [12–14]. MKK4, which plays a vital role in regulating cell signaling induced by cellular stress or inflammatory cytokines, is associated with multiple physiological and pathophysiological processes, such as cardiac hypertrophy, immune response, and cancer development [15–18]. In the previous studies, MKK4 has been shown to exert proapoptotic effects. MKK4 overexpression promotes H_2_O_2_-induced vascular smooth muscle cell apoptosis, and MKK4/JNK signaling pathway inhibition reduces tributyltin chloride-induced apoptosis in hypothalamic neurons [19, 20]. Reactive oxygen species (ROS) accumulation triggers activation of the MKK4/JNK pathway and apoptosis [21]. Baicalein protects mitochondria against H_2_O_2_-induced oxidation by suppressing MKK4/JNK cascades [22]. Moreover, the role of MKK4/JNK in the liver has been investigated. MKK4 is a crucial regulator of liver regeneration, and MKK4 suppression markedly promotes the regenerative capacity of hepatocytes in mouse models [23]. Additionally, MKK4 knockdown significantly protects against acetaminophen-induced histological liver necrosis and inhibits the increase in serum alanine transaminase [24]. In the mouse liver, MKK4 is upregulated at 1 h post-HS [25]. However, the function of MKK4 in HS-induced liver damage is unclear.

MKK4 is essential for liver regeneration and damage repair. Based on these findings, we hypothesize that MKK4 plays a vital role in HS-induced liver injury by regulating apoptosis, oxidative stress, and mitochondrial function in hepatocytes, and this process may involve the JNK/pathway. This study used a rat model of HS to examine the function of MKK4 in liver injury *in vivo* and demonstrated that MKK4 knockdown plays a protective role in HS-induced liver injury by inhibiting JNK/c-Jun activation.

## 2. Materials and Methods

### 2.1. Clinical Study

A clinical study was conducted in 7 patients with severe HS after polytrauma from Wuxi 9th Affiliated Hospital of Soochow University. Thirty healthy participants served as the controls. The blood samples were collected from all subjects after obtaining written informed consent. The study was performed in accordance with the Declaration of Helsinki and approved by the Ethics Committee of the Wuxi 9th Affiliated Hospital of Soochow University.

### 2.2. Hemorrhage Shock Model

**Figure 1:**
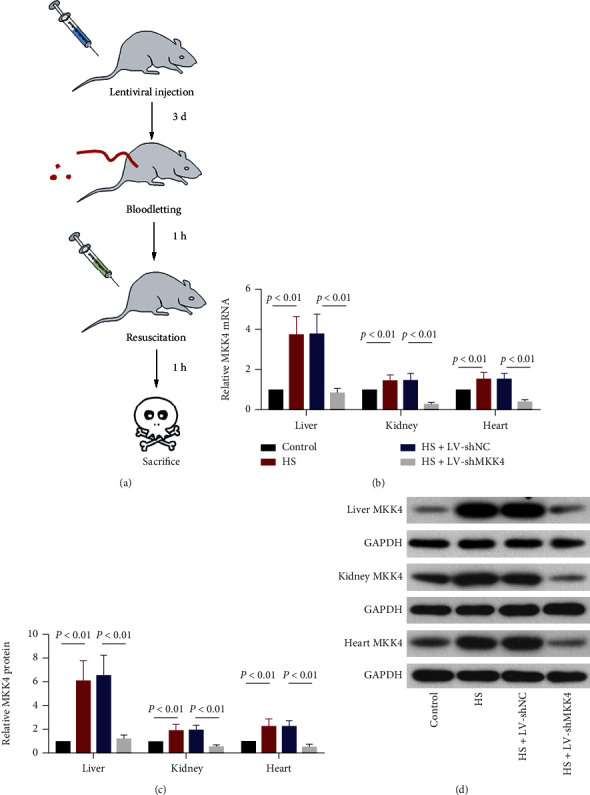
MKK4 expression in rat liver is strongly induced by HS. (a) Schematic illustration of the experimental protocols. The rats were injected with lentivirus encoding shRNA against MKK4 or negative control shRNA. Three days later, the rat models of HS were established. Sprague-Dawley rats were subjected to bloodletting via a catheter inserted into the femoral artery, and then, mean arterial pressure (MAP) was maintained at 40–50 mmHg for 1 h. After 1 h of shock, the rats were resuscitated to restore MAP. One hour later, the rats were sacrificed, and tissues were collected for analysis. (b) The relative mRNA expression of MKK4 in rat liver, kidney, and heart tissues was measured by RT–qPCR. (c, d) Quantification and representative Western blot images of MKK4 protein expression in rat liver, kidney, and heart tissues. The data are expressed as mean ± SD, *n* = 6 for each group.

Twelve-week-old Sprague-Dawley rats obtained from Liaoning Changsheng Biotech Co., Ltd. (Benxi, China) were allowed free access to food and water throughout acclimatization for 1 week. Three days before modeling, rats were injected through the tail vein with 1.0 × 10^8^ transduction units of lentivirus encoding short hairpin RNA (shRNA) against the MKK4 (LV-shMKK4) or negative control shRNA (LV-shNC). HS was induced by bloodletting via a femoral arterial catheter, and the mean arterial pressure (MAP) was maintained at 40-50 mm Hg for 1 h. Rats were resuscitated with lactated Ringer's solution for 1 h. Control rats underwent the same surgical procedure without bloodletting and resuscitation. One hour after resuscitation, all rats were sacrificed. Liver tissues and blood were collected for analysis (Figure 1(a)). All animal experiments were approved by the Ethics Committee of Wuxi 9th Affiliated Hospital of Soochow University (No. KT2020029).

### 2.3. Real-Time PCR

Total RNAs in rat liver were isolated using Total RNA Isolation Kit (Tiangen Biotech Co. Ltd., Beijing, China) and reverse transcribed into cDNA with BeyoRT II M-MLV reverse transcriptase (Beyotime, Shanghai, China). Then, cDNAs were amplified with 2×Taq PCR MasterMix (Solarbio, Beijing, China) and SYBR Green (Solarbio, Beijing, China) under Exicycler™ 96 Real-time PCR System (Bioneer Corporation, Daejeon, Korea). The mRNA expression level was calculated using the 2^-*ΔΔ*Ct^ method and normalized to GAPDH. The primers were synthesized by Genscript Biotechnology Co., Ltd. (Nanjing, China), and the sequences were as follows: MKK4, 5′-CAACACTGGGATTTCACT-3′ (forward), 5′-ACTACTCCGCATCACTACA-3′ (reverse); hypoxia-inducible factor-1*α* (HIF-1*α*), 5′-CTATGTCGCTTTCTTGG-3′ (forward), 5′-TTTCTGCTGCCTTGTAT-3′ (reverse); and GAPDH: 5′-CGGCAAGTTCAACGGCACAG-3′ (forward), 5′-CGCCAGTAGACTCCACGACAT-3′ (reverse).

### 2.4. Western Blot Analysis

The liver tissues from the rats were homogenized in RIPA buffer (Solarbio, Beijing, China), and total proteins were isolated. According to the manufacturer's instructions, liver tissues were cut into pieces, and the proteins in mitochondria were extracted using a mitochondrial protein extraction kit (Jiancheng Bioengineering Institute, Nanjing, China). Protein concentrations were quantified with a BCA kit (Solarbio, Beijing, China). Protein lysates of liver tissues were fractionated on SDS-PAGE and transferred onto polyvinylidene fluoride (PVDF; Millipore, Billerica, MA, USA) membrane. After being blocked in 5% milk (Sangon Biotech, Shanghai, China) and dissolved in TBS-Tween for 1 h, the membrane was incubated with the primary antibodies purchased from Affinity Biosciences (Cincinnati, OH, USA), including antibody to MKK4 (1 : 500 dilution), HIF-1*α* (1 : 500 dilution), p-JNK (Thr183/Tyr185) (1 : 1000 dilution), JNK (1 : 500 dilution), p-c-Jun (1 : 1000 dilution), c-Jun (1 : 500 dilution), Bax (1 : 500 dilution), cleaved caspase 3 (1 : 1000 dilution), Bcl-2 (1 : 500 dilution), and cytochrome c (1 : 500 dilution) overnight at 4°C. Antibody to GAPDH (1 : 10000 dilution; Proteintech Group, Inc., Rosemont, IL, USA) or COX IV (1 : 1000 dilution; Gene Tex, US) was used for reference. Then, the membrane was incubated with the secondary antibody-horseradish peroxidase conjugate (Solarbio, Beijing, China) and developed with an enhanced chemiluminescent (ECL) kit (Solarbio, Beijing, China). Band intensities were quantified and normalized to GAPDH or COX IV.

### 2.5. Histological Analysis and TUNEL

Liver tissues fixed in 4% paraformaldehyde were embedded in paraffin. The tissue block was cut at a thickness of 5 *μ*m. After rehydration, the liver sections were stained with hematoxylin (Solarbio, Beijing, China) and eosin (Sangon Biotech, Shanghai, China). Liver injury was examined by light microscopy (BX53; Olympus, Tokyo, Japan). Apoptosis in the liver was detected using terminal deoxynucleotidyl transferase-mediated dUTP-biotin nick end labeling (TUNEL) staining. Paraffin-embedded liver sections were permeabilized in 0.1% Triton X–100 (Beyotime, Shanghai, China), labeled with a mixture of enzyme solution and label solution, and counterstained with 4′,6-diamidino-2-phenylindole (DAPI; Beyotime, Shanghai, China) according to the instructions in the In Situ Cell Death Detection Kit (Roche, Nutley, NJ, USA). TUNEL-positive cells were observed under a fluorescence microscope (BX53; Olympus, Tokyo, Japan).

### 2.6. Serum Levels of Interleukin- (IL-) 6, Alanine Transaminase (ALT), and Lactate Dehydrogenase (LDH)

Enzyme-linked immunosorbent assay (ELISA) was used for the detection of IL-6 serum level. Serum was separated from the blood, and IL-6 serum level was examined with Rat IL-6 ELISA Kit (LIANKE Biotech., Co., Ltd., Hangzhou, China) following the manufacturer's instructions. The levels of ALT, an indicator of liver damage, and LDH in serum were measured with an ALT kit (Wanleibio, Shenyang, China) and a lactate dehydrogenase assay kit (Jiancheng Bioengineering Institute, Nanjing, China), respectively.

### 2.7. ROS Generation

ROS in the liver was assessed using the DCFH-DA method and a reactive oxygen species assay kit (Jiancheng Bioengineering Institute, Nanjing, China). Liver tissues were cut up for preparing liver cell suspension. Cell suspensions were centrifuged and resuspended in phosphate buffer saline (PBS). Cells were incubated with DCFH-DA, a fluorescent probe for ROS, for 30 min at 37°C. The fluorescence intensity of ROS was detected by a fluorescence microplate reader (Synergy H1; BioTek, Winooski, VT, USA). The excitation wavelength was 490 nm, and the emission wavelength was 530 nm.

### 2.8. Mitochondrial Membrane Potential (MMP)

MMP in the liver was assessed with mitochondrial membrane potential assay kit with JC-1 (Beyotime, Shanghai, China). Before detection, mitochondria in liver cells were isolated with Tissue Mitochondria Isolation Kit (Beyotime, Shanghai, China). Then, mitochondria isolated from cells were resuspended in PBS, incubated with JC-1 staining solution, and detected by a fluorescence microplate reader. MMP was expressed as the ratio of red fluorescence intensity to green fluorescence intensity.

### 2.9. Malondialdehyde (MDA) and Superoxide Dismutase (SOD)

MDA level and SOD activity in the liver cells were examined using an MDA assay kit (Jiancheng Bioengineering Institute, Nanjing, China) and SOD assay kit (Jiancheng Bioengineering Institute, Nanjing, China), respectively. Liver tissues were homogenized in saline and centrifuged. Proteins in supernatant were quantified with a BCA kit (Beyotime, Shanghai, China). Optical density values of MDA and SOD were detected by ultraviolet-visible spectrophotometer (Youke instrument, Shanghai, China) at 532 nm and 550 nm, respectively. MDA levels and SOD activity were calculated according to the standard curves.

### 2.10. ATP and Mitochondrial Respiratory Chain Complexes

The liver tissues were homogenized in saline and centrifuged. Protein concentration in the supernatant was quantified with a BCA (Solarbio, Beijing, China) kit, and ATP level was examined using an ATP assay kit (Jiancheng Bioengineering Institute, Nanjing, China). Optical density was measured under an ultraviolet-visible spectrophotometer at 636 nm. ATP level was calculated according to the standard curves. The activities of mitochondrial respiratory chain complex I and complex III were detected using the corresponding kit (Solarbio, Beijing, China). Complex I and complex III were extracted using the extract buffer in the kit and quantified with BCA kit, respectively, before their activities were measured according to the instructions.

### 2.11. Statistical Analysis

The data were expressed as mean ± standard deviations (SD). GraphPad Prism 7.0 software was used for mean comparison and difference analysis. The values of different groups were compared with one-way analysis of variance (ANOVA) followed by Tukey's test. *P* < 0.05 indicated a significant difference between different groups.

## 3. Results

### 3.1. The Expression of MKK4 Is Increased in the Serum of HS Patients

**Figure 2:**
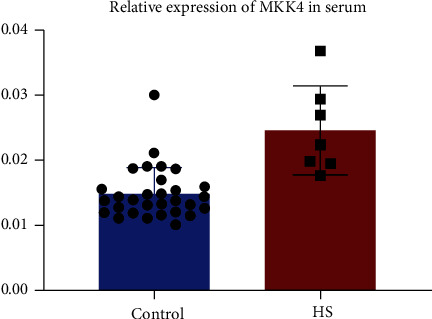
Increased expression of MKK4 in the serum of HS patients. RT-qPCR analysis of the expression of MKK4 in the serum of HS patients (*n* = 7) compared to healthy participants (*n* = 30). Values are expressed as mean ± SD.

The serum samples were collected from 7 HS patients and 30 healthy participants. MKK4 was shown to be highly expressed in the serum of HS patients (Figure 2). Dysregulation of MKK4 in HS patients suggests a potential role of MKK4 in HS pathogenesis, which needs further investigation.

### 3.2. HS Induces MKK4 Upregulation in Rat Liver

To explore the role of MKK4 in HS, a rat model of HS was established, and lentivirus-mediated MKK4 knockdown was carried out in vivo (Figure 1(a)). The expression of MMK4 was examined in the liver and other organs (kidney and heart) of HS rats. The relative expression of MKK4 was significantly increased at both mRNA and protein levels in rat liver, kidney, and heart tissues after HS and resuscitation and was inhibited in all these tissues after injection of LV-shMKK4 via tail vein (Figures 1(b)–1(d)). Taken together, MKK4 expression was upregulated in rat liver after HS and was efficiently knocked down after intravenous injection with LV-shMKK4.

### 3.3. MKK4 Knockdown Mitigates HS-Induced Liver Injury in Rats

**Figure 3:**
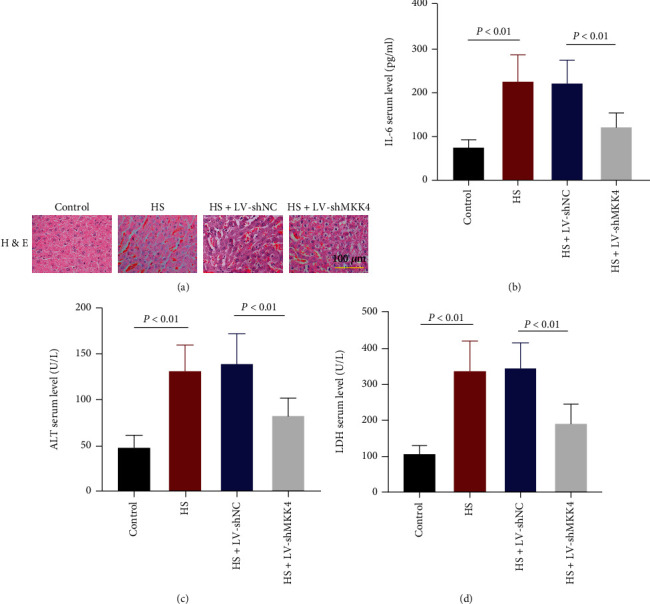
MKK4 knockdown attenuates HS-induced liver injury. (a) H&E staining showed inflammatory infiltration in the livers of rats. Original magnification ×200. Scale bar = 100 *μ*m. (b) The level of IL-6 in the serum of HS model rats was measured by ELISA. (c, d) Alanine aminotransferase (ALT) and lactate dehydrogenase (LDH) levels in the serum of rats were measured one hour after resuscitation. Values are presented as mean ± SD, *n* = 6 for each group.

H&E staining was performed to assess the severity of HS-induced liver injury. Cytoplasmic vacuolization and inflammatory cell infiltration occurred in the liver of HS rats, which was inhibited when MKK4 was knocked down (Figure 3(a)). Additionally, the level of proinflammatory cytokine IL-6 in the serum of rats was detected with ELISA (Figure 3(b)). IL-6 serum level in hemorrhaged rats was markedly elevated after resuscitation compared to control rats, while it was reduced when MKK4 expression was suppressed in hemorrhaged rats. Besides, the serum levels of ALT and LDH, which were increased in HS rats, were decreased due to MKK4 knockdown, indicating that MKK4 knockdown significantly attenuated liver injury induced by HS in the rats (Figures 3(c) and 3(d)).

### 3.4. MKK4 Downregulation Reduces HS-Induced Apoptosis in the Liver

**Figure 4:**
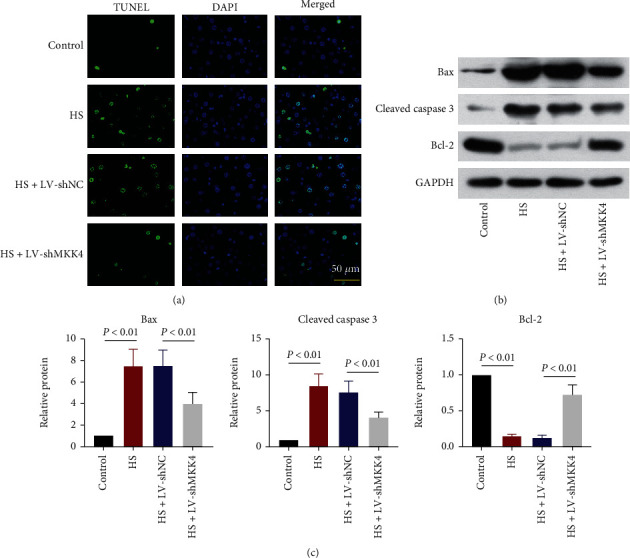
MKK4 downregulation inhibits apoptosis in the livers of HS rats. (a) Representative TUNEL-stained sections of the liver of HS rats. Original magnification ×400. Scale bar = 50 *μ*m. (b, c) Representative Western blot images (b) and quantification of the expression (c) of proteins related to apoptosis, including the proapoptotic factor Bax and cleaved caspase 3 and the antiapoptotic factor Bcl-2. Values are presented as mean ± SD, *n* = 6 for each group.

TUNEL staining and Western blot were performed to investigate MKK4 roles in HS-induced apoptosis in rat liver. The increased number of TUNEL-positive cells in the liver of HS rats compared to control rats showed that HS strongly triggered apoptosis in the liver of rats, while fewer apoptotic cells were detected in the liver of HS rats where MKK4 was knocked down (Figure 4(a)). Bax, cleaved caspase 3, and Bcl-2 are proteins involved in the regulation of apoptosis. The protein levels of proapoptotic factor Bax and cleaved caspase 3 were increased in the liver of HS rats along with the reduced expression of antiapoptotic factor Bcl-2. MKK4 downregulation inhibited the levels of Bax and cleaved caspase 3 and restored Bcl-2 expression in the liver of HS rats, which reflected a marked reduction of HS-induced apoptosis in the liver (Figures 4(b) and 4(c)).

### 3.5. Oxidative Stress Induced by HS in the Liver Is Suppressed by MKK4 Downregulation

**Figure 5:**
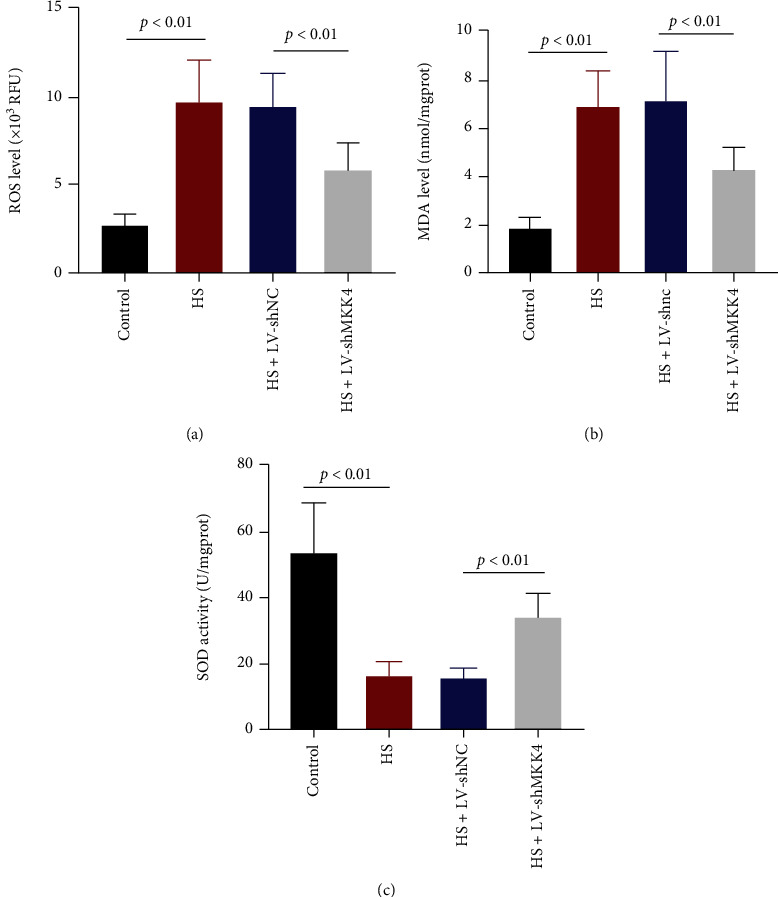
HS-induced oxidative stress in the liver is suppressed when MKK4 is knocked down. (a, b) Reactive oxygen species (ROS) and malondialdehyde (MDA) levels in the livers of HS rats were examined after resuscitation. (c) Superoxide dismutase (SOD) activity in the livers of HS rats was measured. Values are presented as mean ± SD, *n* = 6 for each group.

HS- and resuscitation-induced oxidative stress in the liver was evaluated. Notably, ROS and MDA generation in rat livers was strongly enhanced, while SOD activity was suppressed by HS. When MKK4 was knocked down, the levels of ROS and MDA increased by HS were markedly reduced with elevated SOD activity (Figures 5(a)–5(c)), suggesting that MKK4 may facilitate oxidative stress triggered by HS and resuscitation in rat livers.

### 3.6. MKK4 Knockdown Repairs HS-Induced Mitochondrial Damage and Dysfunction

**Figure 6:**
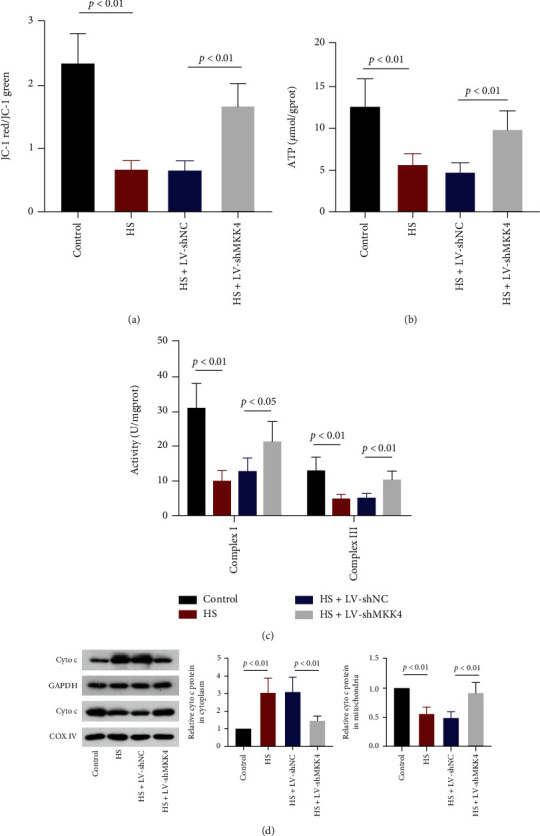
MKK4 knockdown alleviates HS-induced mitochondrial damage in the liver. (a) Changes in mitochondrial membrane potential were examined with JC-1, the fluorescence intensity of red and green was measured, and the ratio was calculated. (b) The level of ATP in the livers of HS rats was measured with a kit. (c) The activities of mitochondrial complex I and complex III in the livers of HS rats were examined. (d) Representative Western blot images and quantification of cytochrome c levels in the cytoplasm and mitochondria. Values are presented as mean ± SD, *n* = 6 for each group.

MMP in liver cells was examined with the JC-1 method. The decreased ratio of JC-1 red fluorescence to green fluorescence in HS rats indicated that HS and resuscitation significantly reduced MMP in the rat liver, which might trigger the reduction of ATP production. MKK4 knockdown partially restored MMP in the liver of HS rats (Figure 6(a)). Consistent with this finding, compared to control rats, a reduction of ATP level in the liver of HS rats was observed, and MKK4 downregulation contributed to the restoration of ATP level (Figure 6(b)). Likewise, the activity of mitochondrial respiratory chain complexes I and III restrained by HS was also restored by MKK4 knockdown in the liver (Figure 6(c)). Besides, cytochrome c in cytoplasm and mitochondria was analyzed. Cytochrome c level in the cytoplasm was increased while that in mitochondria was decreased upon HS. MKK4 downregulation reversed the cytoplasmic or mitochondrial cytochrome c level (Figure 6(d)). This finding indicated that MKK4 knockdown attenuated mitochondrial damage and retracted the release of cytochrome c from mitochondria into cytoplasm caused by HS. Therefore, MKK4 downregulation in the liver prevented mitochondria from HS-induced dysfunction.

### 3.7. MKK4 Knockdown Restricts the JNK/c-Jun and HIF-1*α* Signaling Activation in the Liver

**Figure 7:**
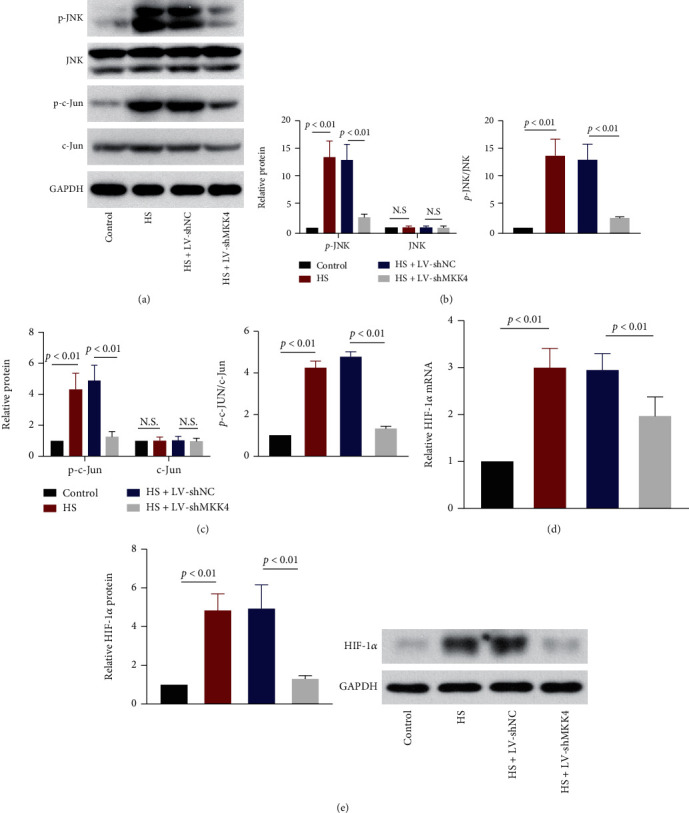
MKK4 downregulation suppresses the JNK/c-JUN and HIF-1*α* pathway. (a) Representative Western blot images of phosphorylated JNK/c-JUN and total JNK/c-JUN in the livers of rats after resuscitation. (b) Quantification of p-JNK and total JNK protein expression and the ratio of these two proteins in rat livers. (c) Quantification of p-c-JUN and total c-JUN expression and the ratio of these two proteins in the livers of HS rats. (d, e) The relative mRNA and protein expressions of HIF-1*α* were detected in rat livers. Values are presented as mean ± SD, *n* = 6 for each group.

To explore the pathway involved in the regulation of MKK4 to HS-induced liver injury, we analyzed the protein levels of JNK and c-Jun. Western blot showed that MKK4 knockdown decreased the phosphorylated JNK levels, which was dramatically increased by HS, without altering the total JNK level, indicating that MKK4 knockdown restricted the phosphorylation and activation of JNK (Figures 7(a) and 7(b)). The same effect of MKK4 knockdown on c-Jun phosphorylation was observed (Figure 7(c)). In addition, HS induced a significant increase in the expression of HIF-1*α*, which was downregulated by knockdown of MKK4 at both mRNA and protein levels (Figures 1(d) and 1(e)). It seemed that the expression of HIF-1*α* was positively correlated with that of MKK4. In this part, MKK4 knockdown was demonstrated to suppress the activation of the JNK/c-Jun and HIF-1*α* signaling pathway.

## 4. Discussion

In this study, we revealed that MKK4 was upregulated in the liver when rats were subjected to HS and resuscitation, and MKK4 knockdown markedly abrogated the massive liver injury induced by HS. Subsequently, we analyzed apoptosis, oxidative stress, and mitochondrial damage in rat livers and found that these responses were significantly inhibited by MKK4 knockdown. Finally, we demonstrated that the JNK/c-Jun pathway in the liver was activated by HS and then inhibited by MKK4 suppression.

HS affects the liver in a bimodal way through ischemia and reperfusion, which stimulates various cell signaling events, exacerbates oxidative stress and inflammatory responses, and ultimately results in hepatocyte death and liver dysfunction [3, 8]. In this study, HS caused the upregulation of MKK4 expression, infiltration of inflammatory cells, and elevation in serum levels of IL-6, ALT, and LDH. MKK4 knockdown reduced hepatocyte fate and the serum levels of these factors, which suggested that MKK4 contributes to the induction of hepatic dysfunction in the context of HS, and MKK4 knockdown could attenuate subsequent liver injury. Similarly, Zhang et al.'s research showed that MKK4 knockdown attenuated the liver injury and ALT elevation in acute murine liver injury models [24]. Therefore, we uncovered the protective effect of MKK4 knockdown against HS-induced liver injury in rats.

However, the mechanism by which MKK4 regulates the development of liver damage in response to HS is largely unclear. Cellular hypoxia during hemorrhage and the sudden increase in oxygen upon resuscitation is considered to be the cause of oxidative stress, thereby inducing mitochondrial damage and subsequent apoptosis [26]. The role of MKK4 in organ injury induced by HS and resuscitation is still poorly understood. A previous study emphasized that inhibiting the MKKE/JNK pathway contributed to apoptosis suppression and liver protection [27]. Consistent with this finding, our results suggested that Bax and cleavage of caspase 3 induced by HS were inhibited in the liver due to MKK4 knockdown, whereas the Bcl-2 expression level was elevated. In addition, HS strongly increased intracellular ROS and MDA generation and restricted SOD activity, indicating that HS enhanced oxidative stress in the liver. Oxidative stress triggered mitochondrial damage and dysfunction, as reflected by decreases in MMP levels, reductions in ATP production and complex I and complex III activity, and promotion of the release of cytochrome c from mitochondria to the cytoplasm. MKK4 knockdown markedly reduced HS-induced oxidative stress and mitigated mitochondrial damage in the rat liver. Here, we demonstrated that MKK4 knockdown exhibited an antioxidative effect on hepatocytes in the context of HS. Inhibiting MKK4/JNK cascades has been reported to significantly suppress Bax expression, caspase 3 activation, the release of cytochrome c from mitochondria into the cytosol, and the production of intracellular ROS. In contrast, inhibiting these cascades increases Bcl-2 levels and restores MMP activity, which indicates that the MKK4/JNK pathway contributes to oxidative stress-induced apoptosis and mitochondrial dysfunction [22, 28]. MKK4 regulates various events in cells by activating JNKs which activate c-Jun expression and phosphorylation [9]. We analyzed the expression of proteins related to the MKK4/JNK pathway, including phosphorylated JNK, total JNK, phosphorylated c-Jun, and total c-Jun. MKK4 knockdown inhibited the phosphorylation and activation of JNK/c-Jun pathway factors.

Hypoxia is a major causative sensor and factor for deleterious oxidative stress in HS, and activation of HIF-1*α* is critically involved in the pathogenesis of liver diseases [29]. HIF-1*α* functions as a key contributor to regulating homeostatic responses to hypoxia and oxidative stresses by activating transcriptional expression of genes that involves in inflammation and cell survival [30, 31]. It is reported that the expression of HIF-1*α* is related to ROS production and oxidative stress in hypoxia-induced rat liver [32]. Hypoxia-inducible factors are considered as molecular target for liver diseases [33]. Inhibition of HIF-1*α* expression or activation protected against experimental liver injury in vivo [34, 35]. HIF-1*α* inhibitor rescued chronic liver failure through inhibiting cellular ROS production and mitochondrial dysfunction [36]. The expression of HIF-1*α* was upregulated at both mRNA and protein levels in liver tissues of rats after HS and resuscitation. Notably, knockdown of MKK4 resulted in a significant downregulation of HIF-1*α*. The expression of HIF-1*α* seemed to be positively correlated with that of MKK4 in HS rats. Suppression of hypoxia-induced activation of JNK signaling including MKK4 inhibited HIF-1*α* expression [37]. MKK4/7-JNK signaling involved in upregulation of HIF-1*α* via activating transcription factor NF-*κ*B [38]. In consistence with the previous studies, knockdown of MKK4 inhibited JNK signaling activation leading to HIF-1*α* downregulation in rat liver induced by HS. It is possible that MKK4 might function through regulating HIF-1*α* expression and its downstream transcriptional events or the relevant signaling pathways in HS. These deserved further investigation for comprehensive understanding of MKK4's role and mechanism in the future.

**Figure 8:**
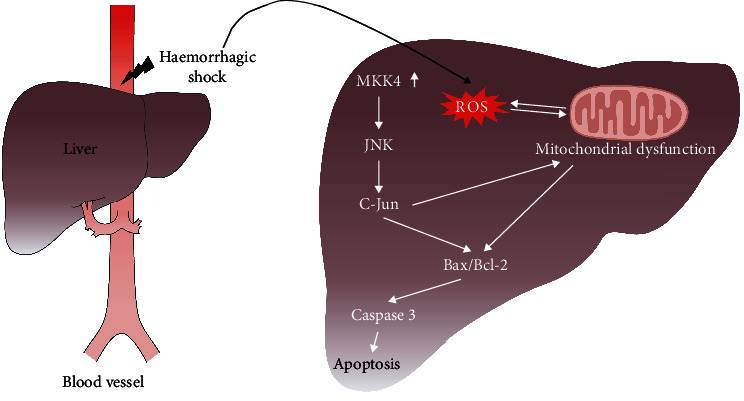
Schematic showing the effects of MKK4 knockdown on HS-induced liver injury. HS induces the upregulation of MKK4 expression and high levels of ROS production in the liver. ROS in the liver cause mitochondrial damage and dysfunction under oxidative stress, which regulates the Bax/Bcl-2 system and induces apoptosis. Moreover, MKK4 upregulation activates the JNK/c-Jun cascade, contributing to Bcl-2 inhibition, Bax activation, and the induction of mitochondrial dysfunction, thereby enhancing ROS generation and inducing apoptosis in the liver. MKK4 may contribute to the development of HS-induced liver injury by activating the JNK/c-Jun pathway.

Overall, HS and resuscitation induce MKK4 expression and cause oxidative stress in the liver. On the one hand, massive amounts of ROS are produced during this process, enhancing the magnitude and duration of JNK activation by suppressing the activity of JNK phosphatases [39]. Prolonged activation of JNK can activate proapoptotic proteins and inhibit antiapoptotic proteins. Bcl-2 is an antiapoptotic factor that is localized in the mitochondrial inner membrane. The proapoptotic protein Bax is localized in the cytosol, which translocates to mitochondria and promotes the release of cytochrome c from the mitochondria into the cytosol, thereby inducing apoptosis [40, 41]. Mitochondria are not only subjected to direct attack by ROS but are also the primary source of ROS production. Mitochondrial dysfunction can also increase ROS production, which increases oxidative stress, disrupts the Bcl-2/Bax balance, and induces apoptosis [28, 42]. On the other hand, HS upregulates MKK4 expression in the liver, contributing to the activation of JNK and c-Jun. MKK4 and JNK rapidly translocate to mitochondria in response to oxidative stress and enhance oxidative stress [43]. Therefore, both HS-induced ROS generation and MKK4 upregulation activate the JNK cascade, which exacerbates oxidative stress and mediates mitochondrial dysfunction, thus inducing apoptosis in the liver. MKK4 knockdown leads to the suppression of JNK activation and inhibits downstream responses in the liver, including mitochondrial damage, oxidative stress, and apoptosis (Figure 8). Here, we preliminarily investigated MKK4 function in HS-induced liver injury and the mechanism involved in this process. However, to make the findings in this study more convincing, further investigation needs to be carried out using hepatocytes to verify these findings *in vitro*.

A potential limitation of the current study is that the loss-of-function study for MKK4 was carried out with the use of lentivirus-mediated RNAi delivered systemically *in vivo*. Except for the liver, the expression of MKK4 was examined in other organs including kidney and heart of HS rats after injection of LV-shMKK4 via tail vein. MKK4 expression was significantly increased in all these organs of HS rats and was efficiently downregulated after systemic delivery of LV-shMKK4. In the current research, the way of RNAi delivered systemically is widely used for the loss-of-function studies for a gene in experimental animal models, including HS or liver injury [44–47]. We here focused on the influence on the liver and aimed to investigate the role of MKK4 in HS-induced liver injury but failed to study the potential side effects on other organs resulted from systemic knockdown of MKK4. The present study together with the previous findings suggest that MKK4 likely exerts deleterious functions in the liver under HS, and HS-induced upregulation of MKK4 may contribute to the development of liver injury. However, the role of MKK4 in HS-induced other organ injury or other pathological events in the liver remains unknown. Whether inhibition of MKK4 breaks the self-protection and brings some side effects is hard to determine according to our preliminary study and deserved more investigation. Also, it is deserved to use RNAi delivered system targeting the liver or conditional knockout mice for better understanding of MKK4's role in HS-induced liver injury. An in-depth exploration and comprehensive understanding of MKK4's role in HS is necessary to be carried out in the future.

In summary, our study identified MKK4 as a critical regulator of HS-induced liver injury. MKK4 knockdown prevented HS-mediated damage to the liver by inhibiting oxidative stress, mitochondrial dysfunction, and apoptosis. We demonstrated that MKK4 may play a protective role in liver injury in response to HS by inhibiting the JNK pathway. The MKK4-JNK pathway may be a therapeutic target for HS-induced liver injury.

## Data Availability

The data sets used and analyzed during this study are available from the corresponding author upon reasonable request.

## Abbreviations

HS: Hemorrhagic shock
JNK: c-Jun NH2-terminal kinase
MKK4: Mitogen-activated protein kinase kinase 4
ROS: Reactive oxygen species
MDA: Malondialdehyde
SOD: Superoxide dismutase
MMP: Mitochondrial membrane potential
ALT: Alanine transaminase
LDH: Lactate dehydrogenase.

## Additional Points

*Open Access*. This article is licensed under a Creative Commons Attribution 4.0 International License, which permits use, sharing, adaptation, distribution, and reproduction in any medium or format, if you give appropriate credit to the original author(s) and the source, provide a link to the Creative Commons license, and indicate if changes were made. The images or other third-party material in this article are included in the article's Creative Commons license, unless indicated otherwise in a credit line to the material. If material is not included in the article's Creative Commons license and your intended use is not permitted by statutory regulation or exceeds the permitted use, you will need to obtain permission directly from the copyright holder. To view a copy of this license, visit http://creativecommons. org/licenses/by/4.0/.
